# Mechanism of action study to evaluate the effect of rosiglitazone on bone in postmenopausal women with type 2 diabetes mellitus: rationale, study design and baseline characteristics

**DOI:** 10.3109/21556660.2011.641703

**Published:** 2011-12-16

**Authors:** Lorraine A. Fitzpatrick, John P. Bilezikian, Margaret Wooddell, Gitanjali Paul, Nikheel S. Kolatkar, Antonio J. Nino, Colin G. Miller, Cesar E. Bogado, Claude D. Arnaud, Alexander R. Cobitz

**Affiliations:** 1 GlaxoSmithKline Research and Development, 709 Swedeland Road, King of Prussia, PA 19406USA; 2 College of Physicians and Surgeons, Columbia University, New York, NY, USA. Department of Medicine, College of Physicians and Surgeons, Columbia University, 630 W. 168th Street, New York, NY 10032USA; 3 GlaxoSmithKline Research and Development, 2301 Renaissance Boulevard, King of Prussia, PA 19406USA; 4 BioClinica Inc., 826 Newtown-Yardley Road, Newtown, PA 18940USA; 5 Instituto de Investigaciones, Metabolicas, Libertad 836 C1012AAR Buenos AiresArgentina; 6 Imaging Therapeutics, 24301 Southland Drive, Suite 623, Hayward, CA 94545USA

**Keywords:** rosiglitazone, metformin, type 2 diabetes mellitus, mechanism of action, bone mineral density, DXA

## Abstract

**Objectives:**

Post-hoc analyses have shown an increase incidence of fractures among type 2 diabetes (T2DM) patients treated with thiazolidinediones (TZDs). The mechanisms by which TZDs may be associated with increased fracture risk is not well understood.

This article describes the study design and baseline characteristics for a prospective, randomized, double-blind, active-controlled trial to evaluate the effects of rosiglitazone on changes in measures of skeletal structure, surrogates of bone strength and metabolism.

**Methods:**

Postmenopausal women without osteoporosis and diagnosed with T2DM were randomized in a double-blind design to either rosiglitazone or metformin for 52 weeks, then all subjects received open-label metformin for 24 weeks. Study endpoints included changes in bone mineral density (BMD), quantitative computed tomography (QCT), digitized hip radiography (HXR) and high resolution magnetic resonance imaging (hrMRI). Serum markers of bone metabolism and indices of glycemic control were assessed within and between treatment groups.

**Results:**

A total of 226 subjects were randomized. Baseline characteristics included: age 63.8 ± 6.5 years; years postmenopausal 16.9 ± 8.4; duration of diabetes 3.5 (1.8–7.8) years; body mass index (BMI) 31.4 ± 5.9 kg/m^2^; and glycated hemoglobin (HbA1c) 6.4 ± 0.65%. At baseline, mean T-scores were −0.95 ± 0.91 at the femoral neck, −0.02 ± 0.97 at the total hip and −0.55 ± 1.25 at the total spine.

Since there are no well recognized techniques to determine bone mass and structure at the distal limbs (cortical bone sites where fractures were reported in RSG subjects), using the femoral neck as a surrogate for these areas may be a potential limitation of the study.

**Conclusion:**

This is the first randomized trial utilizing multiple techniques to evaluate bone mass, structure, serum markers of bone remodeling, and potential reversibility of changes after discontinuation of rosiglitazone. This study will provide information about RSG bone effects in a population of postmenopausal women at risk for bone loss and subsequent fracture.

**ClinicalTrials.gov number:**

NCT00679939

## Introduction

Postmenopausal women are at higher risk of osteoporosis and subsequent fractures than premenopausal women and men, making them theoretically a more vulnerable population to interactions with concomitant risk factors for fractures. Evidence suggests that women with type 2 diabetes mellitus (T2DM) have normal or higher bone mineral density (BMD) but approximately double the overall risk of skeletal fractures when compared with non-diabetic subjects.^1^^–^^5^ In the Women’s Health Initiative Observational Cohort, BMD was significantly higher at the spine and hip in diabetic women compared with control subjects. The overall risk of fractures was higher than in non-diabetics, after controlling for multiple factors, including frequency of falls. The risk of fracture of the hip/pelvis/upper leg was also increased. In the Study of Osteoporotic Fractures, older women with diabetes were found to have 30% higher risk of non-vertebral fractures in comparison with non-diabetics. Information in different populations corroborates T2DM in women as an independent risk factor for fractures.^6,7^

The actions of pharmacologic treatments for T2DM on fracture risk in postmenopausal women are poorly defined. In one study, metformin (MET) was associated with decreased fracture risk, whereas in another, the use of MET was not associated with any effects.^8^ The thiazolidinediones (TZDs) rosiglitazone (RSG) and pioglitazone (PIO) have been associated with an increased risk of skeletal fractures. In ADOPT (A Diabetes Outcomes Progression Trial), a post-hoc analysis indicated that postmenopausal women treated with RSG experienced an increased risk of fractures in comparison with patients receiving MET or glyburide, with the majority of fractures reported in the upper extremity and foot.^9^ In the RECORD (Rosiglitazone Evaluated for Cardiovascular Outcomes and Regulation of Glycaemia in Diabetes) study, this finding was confirmed with the analysis of self-reported adverse events.^10^ Similarly, increased incidence of distal extremity fractures in women receiving long-term treatments with PIO for T2DM was reported in a post-hoc analysis of the PROactive (Prospective Pioglitazone Clinical Trial in Macrovascular Events) trial.^11,12^

The purpose of this study was to understand the underlying mechanisms that could account for the increased fracture risk in T2DM subjects taking RSG as these fractures are not typically seen in osteoporotic, postmenopausal women. In addition, the loss of bone mass demonstrated in preclinical and clinical studies does not explain the increase in fracture at these atypical sites. Since understanding the clinical significance of this apparent increase in fracture risk remains incomplete, the need for additional data to elucidate mechanisms by which RSG may affect bone health is necessary.

The complexity of the study with multiple types of image acquisitions, the use of a novel digitized hip X-ray radiographic technique and the examination of QCT images by quadrant justifies this separate report of the study design and baseline characteristics so these important details can be adequately described.

## Methods

### Study design

**Figure 1.**
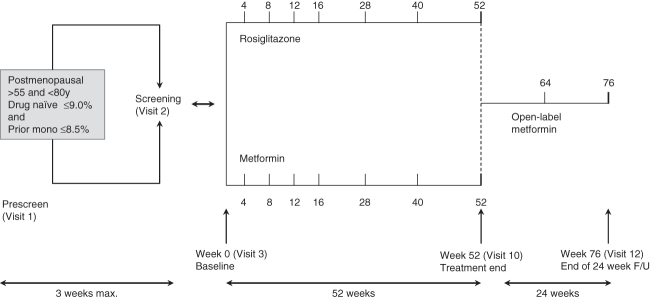
Study design. For pre-screening HbA1c, if subject is drug-naïve, the pre-screening HbA1c ≤9.0%. If prior monotherapy, subject on maximal doses of metformin (≤1000 mg MET), sulfonylureas, (≤5 mg glyburide, ≤10 mg glipizide or ≤8 mg glimepiride) or full-dose sitagliptin (100 mg sitagliptin) has a pre-screening HbA1c ≤8.5% OR if subject on greater than submaximal doses of metformin (>1000 mg) or sulfonylureas (>5 mg glyburide, >10 mg glipizide or >8 mg glimepiride), their pre-screening HbA1c ≤7.0%.

This double-blind, randomized, multinational, active-controlled trial, was divided into 3 phases: screening, 52-week double-blind treatment with RSG or MET, and a 24-week open-label follow-up with all subjects receiving MET (Figure 1). The protocol (GSK study number AVD111179) is registered on ClinicalTrials.gov as NCT00679939.

Investigational Review Board approval was obtained and all subjects provided written informed consent prior to any trial assessments being performed. The primary end point was percentage change in BMD by dual energy X-ray absorptiometry (DXA) at the femoral neck from baseline to Week 52 in the RSG treatment group. Additional determinations made at the lumbar spine, total hip and trochanter from baseline to Weeks 16, 28, 52 and 76 within the RSG treatment groups will be analyzed. Additional assessments include drug effects on trabecular and cortical BMD and structural analysis by quantitative computed tomography (QCT), radiographic anatomy by digitized hip radiography (HXR), and micro- and macro-architecture by wrist high resolution magnetic resonance imaging (hrMRI) within and between the RSG and MET groups. Changes in serum markers of bone remodeling, namely, bone-specific alkaline phosphatase (BSAP), carboxyterminal cross-linked telopeptide of Type I collagen (CTX) and procollagen type 1 N-propeptide (P1NP) are being measured. In addition, serum calcium (Ca), 25-hydroxy vitamin D, parathyroid hormone (PTH), serum total and free testosterone, estradiol, and sex hormone binding globulin (SHBG) were monitored. Secondary and tertiary objectives include the evaluation, within and between the treatment groups, of change from baseline in BMD by DXA and QCT, HXR and hrMRI, at select time points and anatomical sites, glycated hemoglobin (HbA1c), fasting plasma glucose (FPG) and insulin, serum biomarkers of bone remodeling, calcium homeostasis and select sex hormone changes over time.

### Participants

Women from 55 to 80 years of age, >5 years postmenopausal, with a diagnosis of T2DM and prior antidiabetic therapy with diet and exercise alone (drug-naive) or prior monotherapy (non-TZD) for more than 2 weeks in the past 12 weeks were eligible for enrolment in the study. Furthermore, subjects had to weigh less than 136.4 kg, and have a BMD T-score >−2.5 at the femoral neck, total hip and lumbar spine. At screening, HbA1c had to be ≤9% for drug-naive subjects; ≤8.5% for those on submaximal doses of oral antidiabetic agents and ≤7.0% for subjects on maximal doses.

The main exclusion criteria included type 1 diabetes mellitus, diabetic ketoacidosis, uncontrolled hypertension, cancer in the past 5 years (except previously selected treated skin cancers), drug or alcohol abuse, simultaneous treatment with ≥2 antidiabetic agents in the past 12 weeks, significant renal or hepatic diseases, severe edema associated with TZD use, heart failure, anemia, macular edema, and active coronary heart disease. Skeletal and mineral metabolism exclusion criteria included bilateral hip replacements, diseases affecting bone metabolism, active nephrolithiasis, and abnormal serum calcium level. Chronic use of systemic corticosteroids and previous treatment with bone active drugs, contraindications to therapy with calcium or vitamin D, MET or RSG, were exclusionary.

To obtain similar glycemic control between treatment groups, a titration algorithm of study medications was used and the study was controlled for concomitant antidiabetic medications used after randomization. At baseline, subjects on prior monotherapy were switched to study medication. RSG was started at a total daily dose of 4 mg and force-titrated to 8 mg. MET was begun at 1000 mg and force-titrated to 2000 mg. From Weeks 8 to 16, subjects with mean daily glucose (MDG) >110 mg/dl at the maximum tolerated doses of blinded RSG or MET received open label sulfonylurea (SU). After 4 months of double-blind treatment, subjects with HbA1c >7.5% at the maximum dose of double-blind study medication could up-titrate or add open-label SU therapy at the discretion of the investigator. After 52 weeks, subjects were force-titrated to a total daily dose of 2000 mg MET in an open-label manner. Subjects with poor glycemic control at the maximum tolerated dose of MET were up-titrated to additional open-label SU at the discretion of the investigator. Precautions to attenuate the risk of fractures during the study included: exclusion of patients with a history of osteoporosis; providing all subjects with daily supplementation of calcium (500–1000 mg) and vitamin D (at least 400 IU daily); and central monitoring of BMD by DXA. If there was a BMD decrease of >6.0% at the lumbar spine or total hip or >7.0% at the femoral neck at the 7- and 12-month intervals, a confirmatory DXA scan was acquired; if the results confirmed the initial findings, the results were provided to the investigator to be discussed with the subject for appropriate management.

### Dual Energy X-ray absorptiometry

Areal BMD was measured by DXA instruments manufactured by Hologic Inc. (Bedford, Massachusetts, United States) or GE Healthcare Lunar (Madison, Wisconsin, United States). At screening, subjects had DXA scans of the left femoral neck, total hip and posterior-anterior L1-L4 lumbar spine. A minimum of 3 evaluable vertebrae were required at baseline. Each subject was measured on the same DXA instrument throughout the study. Standardized procedures for evaluating monthly instrument quality control, including qualifying instrument quality control to ensure stable instrument calibration prior to enrolling subjects were conducted. Cross-calibration of the scanners was evaluated using the Bona Fide Phantom (BioClinica Inc., Newtown, Pennsylvania, United States) which was further used for calculation of any shifts in instrument calibration. Subject DXA scans were performed also at Weeks 16, 28, 52 and 76. To be eligible, the absolute BMD values consistent with a T-score at the femoral neck, lumbar spine and total hip >−2.5 was required based on the NHANES database of a normal population. All DXA exams were sent to a central reading facility for quality control and analysis (BioClinica Inc. Newtown, Pennsylvania, United States; or Leiden, the Netherlands).

### Hip Structural Analysis

Hip Structural Analysis (HSA) parameters were measured by DXA at baseline and Weeks 52 and 76 in a subset of subject scans acquired on scanners manufactured by Hologic Inc. Specific measurements include regional areas, bone mineral content (BMC), BMD, T-scores and Z-scores, cortical areas, hip axis length. HSA cross-sectional dimensions were used to analyze the geometry of three femoral regions providing buckling ratio, modulus Z, neck shaft angle, cortex thickness, cross sectional moment of inertia (CSMI), cross-sectional areas (CSA), and endocortical width. The mechanical strength of the hip was estimated based on comparisons of geometric effects between the proximal femur shaft, assumed a purely cortical structure, and the narrow-neck and intertrochanteric regions, assumed mixed cortical/trabecular structures.^13^

### Proximal femur radiographs

**Figure 2.**
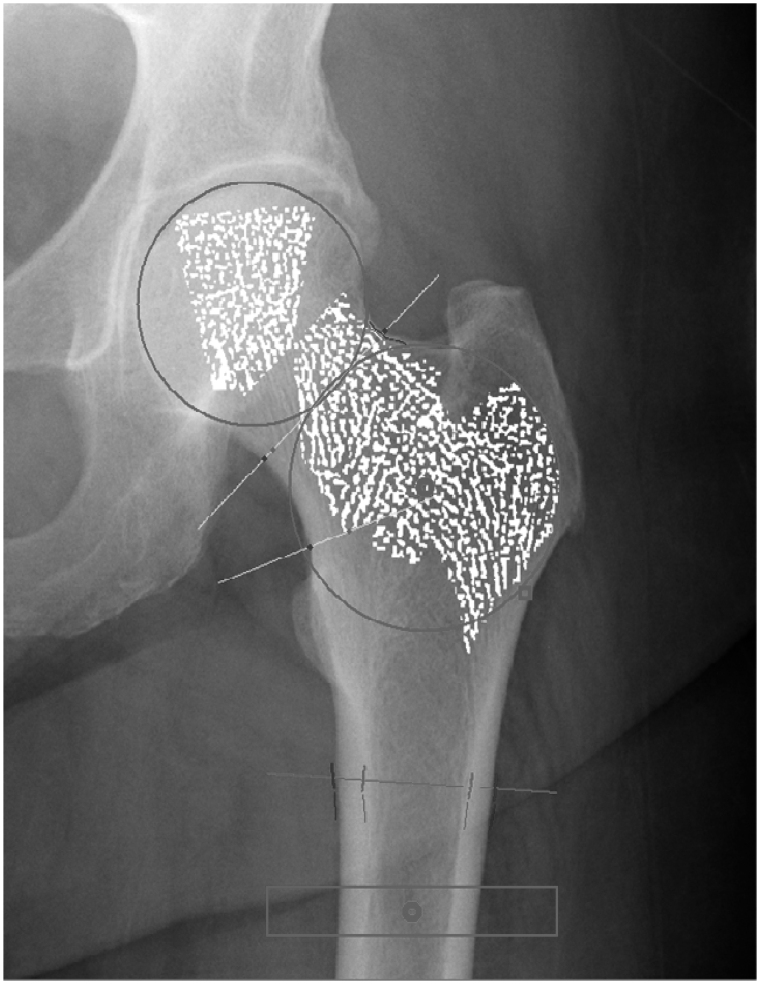
Example Hip X-ray image. Example X-ray image from one of the cases enrolled in the study. Cortical thickness measurements are shown by overlaying red-blue markers placed at several locations along the cortical bone boundary. The highlighted structures on the femoral head, neck and trochanteric regions correspond to the underlying projected bone structures (trabeculae) extracted for further analysis and measurements.

The study will explore if any of the structural bone measurements performed by Imaging Therapeutics, Inc. (ImaTx) software tools are significant in showing changes between measurements performed on hip radiographs taken at baseline and at 52- and 76-week follow-up. Measurements were performed from each available hip radiographic image using ImaTx’s software tools. The measurements target the general morphology of the proximal femur bone, as well as properties of the cortical bone and the projected internal bone structure (bone trabeculae) such as structure length, thickness, spacing, connectivity and fragmentation. A total of 30 femoral geometry and cortical macro-anatomical-parameters were measured including hip axis length, neck-shaft angle, neck cortical thickness and shaft cortical thickness. Standardized general processing steps were implemented for each radiograph, the software was semi-automatically placed over nine regions of interest in the proximal femur radiograph and all subsequent image processing and measurement steps were fully automated. Figure 2 shows an example of a hip X-ray with an overlay of the corresponding regions of interest and extracted bone structures from which all measurements were derived. Measures of micro-anatomical parameters were estimated on segmented dominant structures of projected trabecular patterns. The extracted dominant structures were processed by skeletonization to yield simplified maps of the structures within each region. The skeletonized dominant structures were used to obtain measurements of structure dimension, length, width and thickness, as well as structure distribution and fragmentation, number of nodes and loops, structure intensity distribution, and the range and values for intensity. Algorithms that measure a composite of femoral geometry, cortical dimensions, and trabecular parameters in projection radiographs were used, as described elsewhere.^14^ For each of the nine regions of interest (ROI) defined over the proximal femur image area, 26 bone parameters were derived.

### Quantitative computed tomography

QCT scans of the L1-L3 lumbar spine and both hips were acquired at baseline, Week 52 and Week 76. The hip scans, acquisition protocol required a ROI from a point extended from just above the femoral head to at least 1 cm below the lesser trochanter. Subjects were scanned using the standard protocol with an in-scan phantom with known hydroxyapatite concentrations (Mindways Software Inc., Austin, Texas, United States) to calibrate CT values to BMD. To monitor the CT system performance throughout the study, a Mindways QA phantom was scanned in conjunction with the calibration phantom in accordance with the standard prescribed procedures defined by Mindways. Quality review and QCT scan analysis occurred centrally at an image reading facility (BioClinica Inc., Newtown, Pennsylvania, United States). QCT scans were analyzed using the QCTPro™ (version 4.1.3) system from Mindways Software Inc. From the lumbar spine analysis, average volumetric BMD was calculated. Areal and volumetric BMD, bone mineral content and bone volume for integral bone and trabecular and cortical bone separately, were obtained for total hip, femoral neck and the trochanteric and intra-trochanteric regions from the hip QCT scans.

### High-resolution MRI

HrMRI imaging of trabecular microarchitecture of the distal radius was performed on a 1.5 T Signa system (General Electric, Milwaukee, Wisconsin, United States) using a 4-channel phased array high definition wrist coil (General Electric, Milwaukee, Wisconsin, United States). MRI images were electronically transferred to the MRI Analysis Center, University of California, San Francisco and images were analyzed using in-house image analysis software. Previously described methods were used to compute the apparent trabecular structural parameters such as apparent bone-volume over total-volume fraction (app.BV/TV), apparent trabecular plate separation (app.TbSp), apparent trabecular plate thickness (app.TbTh) and apparent trabecular plate number (app.TbN).^15^ For cortical bone measurements, a direct distance transformation method was used in 3D (DT3D). This method uses growing spheres within the cortical region and outputs the average cortical thickness. The cortical area was determined by counting the pixels in the segmented cortical region.^16^

### Clinical laboratory

Clinical laboratory assessments included: HbA1c (Tosoh Corp., ion exchange HPLC), FPG (Olympus AU2700/5400), plasma insulin (Linco RIA) and serum insulin-like growth factor-1 (IGF-1) (Siemens DPC Immulite, chemiluminescence), BSAP (chemiluminescence immunoassay/Beckman Access), CTX (electrochemiluminescence immunoassay, Roche Elecsys), P1NP radioimmunoassay (Orion Diagnostica RIA Kit), corrected serum calcium (Olympus AU2700/5400 calculated as [(4 - serum albumin g/dl) * 0.8 +serum calcium mg/dl]), and 25-hydroxyvitamin D (liquid chromatography tandem mass spectrometry), parathyroid hormone (PTH) (DPC Immulite 2000 intact PTH assay), serum estradiol (Quest Laboratory, LC/MS/MS), total testosterone (Quest Laboratory, LC/MS/MS), free testosterone (tracer equilibrium dialysis, calculation) and SHBG (Siemens DPC Immulite, chemiluminescence).

### Statistical analyses

This study utilized a computer-generated central randomization within each geographical region stratified by prior therapy (drug-naive and prior monotherapy) and randomized in a 1:1 ratio to one of two treatment arms (RSG and MET). Subjects were registered and medication was ordered using an interactive voice response system. The sample size calculation was based on a 30% drop-out rate and a standard deviation (SD) of 4% for percent change from baseline in femoral neck ensuring that the 95% confidence interval (CI) will be the mean ±0.9% for each treatment group. Treatment differences at 52 weeks for change from baseline for selected parameters will be assessed by an analysis of covariance with terms for treatment, baseline value, prior therapy and region. The safety population, comprising all subjects who had received at least one dose of drug, was used for analysis of all parameters. For these baseline data values, statistical significance had not been tested and therefore, p values do not apply.

## Results

A total of 316 subjects were screened in 39 investigational sites in 8 countries (Argentina, Canada, Estonia, Mexico, Pakistan, Philippines, Spain, and United States). A total of 226 subjects were randomized into the study (randomized population) with 225 subjects receiving at least one dose of the study medication. The demographics are presented in Table 1. Mean age was 63.8 ± 6.5 years and the mean number of years postmenopausal was 16.9 ± 8.4 years. Fifty-four percent of the subjects reported Hispanic or Latino ethnicity and the geographic ancestry distribution for the whole population was Caucasian 71.1%, African-American 4.4%, Asian 17.3% and Other 7.1%. In total, 14.7% of subjects were past or current smokers and 16.9% were alcohol consumers. The median (interquartile range) duration of diabetes was 3.5 (1.8–7.8) years; and 27.6% of the participants were previously treated with diet and exercise alone, the remaining subjects were receiving oral antidiabetic monotherapy. The more prevalent self-reported diabetes complications at enrolment were peripheral neuropathy 7 (3%), diabetic retinopathy 2 (<1%), diabetic foot ulcer 1 (<1%) and microalbuminuria 1 (<1%). The most prevalent general medical conditions were dyslipidemia 105 (47%), hypertension 159 (71%), cardiac arrhythmia 10 (4%), and cerebrovascular disease 6 (3%). The most frequently prescribed prior treatments for concomitant conditions were calcium supplements (calcium with or without vitamin D) 31 (13.8%), vitamin D metabolites or analogues (4.0%), angiotensin-converting enzyme/angiotensin receptor blockers (ACEIs/ARBs) 119 (52.9%), statins 87 (38.7%) and aspirin 54 (24.0%). MET was administered to 121 (53.8%), SUs to 23 (10.2%) and DPP4 inhibitor to 1 (0.4%) of the subjects prior to baseline.

**Table 1.** Baseline demographic characteristics and medications.

	*N* = 225
Age, years, mean (SD)	63.8 (6.5)
Years postmenopausal, mean (SD)	16.9 (8.4)
Self-reported ethnicity, *n* (%)	
Hispanic or Latino	122 (54.2%)
Not Hispanic or Latino	103 (45.8%)
Self-reported geographic ancestry, *n* (%)	
Caucasian	160 (71.1%)
African-American	10 (4.4%)
Asian	39 (17.3%)
Other	16 (7.1%)
Smoking status, *n* (%)	
Current	9 (4.0%)
Past	24 (10.7%)
Never	192 (85.3%)
Duration of diabetes, years, median (IQR)	3.5 (1.8, 7.8)
Baseline diabetes therapy, *n* (%)	
Diet and exercise alone	62 (27.6%)
Oral monotherapy	163 (72.4%)
Self reported diabetes complications, *n* (%)	
Diabetic retinopathy	2 (<1%)
Peripheral neuropathy	7 (3%)
Dyslipidemia	105 (47%)
Diabetic foot ulcer	1 (<1%)
Microalbuminuria	1 (<1%)
Hypertension, *n* (%)	159 (71%)
Prior medication use, *n* (%)	
Aspirin	54 (24.0%)
Beta-blockers	48 (21.3%)
ACEI/ARBs	119 (52.9%)
Statins	87 (38.7%)
Fibrates or other non-statin LLA	25 (11.1%)
Other antiplatelet	6 (2.7%)
Oral calcium with/without vitamin D	31 (13.8%)
Vitamin D metabolites and analogues (no calcium)	9 (4.0%)
NSAIDS	15 (6.7%)
Glucocorticoid, except cream	4 (1.8%)
Calcium channel blocker	41 (18.2%)
Levothyroxine	17 (7.6%)
OAD sulfonylurea	23 (10.2%)
OAD metformin	121 (53.8%)
OAD DPP4 Inhibitor	1 (0.4%)

Notes: SD = standard deviation; IQR = interquartile range; ACEI/ARBs = angiotensin-converting. Enzyme/angiotensin receptor blockers; LLA = lipid lowering agent; NSAIDs = non-steroidal anti-inflammatory drugs. OAD = oral antidiabetic agent; DPP4 = dipeptidyl peptidase-4-inhibitor.

At baseline, mean femoral neck BMD was 0.83 g/cm^2^, mean spine BMD was 1.05 g/cm^2^, and total hip BMD 0.98 g/cm^2^. The corresponding mean femoral neck, spine and total hip T-scores were −0.95 ± 0.91, −0.55 ± 1.25 and −0.02 ± 0.97, respectively. Median Baseline Composite Parameter based on both micro- and macro-measurements measured from baseline X-ray images was 6.1 (2.7 – 9.1).

Baseline skeletal and glycemic indices are presented in Table 2. Physical characteristics (mean ± SD or median [interquartile range]) included BMI 31.4 ± 5.9 kg/m^2^, hip circumference 109.3 ± 13.0 cm, waist circumference 99.3 ± 11.9 cm, and waist/hip ratio 0.9 ± 0.08. Blood pressure indices included systolic 129.7 ± 11.8 and diastolic blood 76.4 ± 7.3 mmHg pressure. Biochemical markers of bone turnover included BSAP 12.5 (10.1–15.0) mcg/l; P1NP 33 (25–42) mcg/l; and CTX 284 (206–399) pg/ml. Indices reflecting mineral metabolism included 25-hydroxyvitamin D: 75 (57–95) nmol/l and serum calcium: 2.4 ± 0.09 mmol/l. Hormones measures included total estradiol 51 (22– 84) pmol/l; total testosterone 0.6 (0.4–0.9) nmol/l; free testosterone 1.0 (0.8–1.3) %, SHBG 35 (24–47) nmol/l, and IGF-1: 13.1 ± 4.8 nmol/l. HbA1c and FPG baseline levels were 6.4 ± 0.7% and 6.2 ± 1.3 mM/l, respectively.

**Table 2.** Baseline physical examination, laboratory and image assessments.

	*N* = 225
Weight, kg, mean (SD)	76.9 (16.0)
BMI kg/m^2^, mean (SD)	31.4 (5.9)
Systolic blood pressure, mm Hg, mean (SD)	129.7 (11.8)
Diastolic blood pressure, mm Hg, mean (SD)	76.4 (7.3)
Hip circumference, cm, mean (SD)	109.3 (13.0)
Waist circumference, cm, mean (SD)	99.3 (11.9)
Waist/hip ratio, mean (SD)	0.91 (0.08)
HbA1c, %, mean (SD)	6.4 (0.65)
FPG, mmol/l, mean (SD)	6.2 (1.3)
IGF-I, nmol/l, mean (SD)	13.1 (4.8)
BSAP, mcg/l, median (IQR)	12.5 (10.1. 15.0)
PINP, mcg/l, median (IQR)	33.0 (25.0, 42.0)
CTX, pg/ml, median (IQR)	284.0 (206.0, 399.0)
25 hydroxy vitamin D, nmol/L, median (IQR)	75.0 (56.5, 95.0)
PTH, ng/l, median (IQR)	36.5 (27.0, 52.0)
Serum Ca, mmol/l, mean (SD)	2.4 (0.09)
Serum estradiol, pmo/l, median (IQR)	51.0 (22.0, 84.0)
Total testosterone, nmol/l, median (IQR)	0.6 (0.4, 0.9)
Free testosterone, %, median (IQR)	1.0 (0.8, 1.3)
SHBG, nmol/l, median (IQR)	34.5 (24.0, 47.0)
Femoral neck BMD, g/cm^2^, mean (SD)	0.83 (0.141)
Total hip BMD, g/cm^2^, mean (SD)	0.98 (0.123)
Total spine BMD, g/cm^2^, mean (SD)	1.05 (0.161)
Femoral neck T-score, mean (SD)	−0.95 (0.91)
Total hip T-score, mean (SD)	−0.02 (0.97)
Total spine T-score, mean (SD)	−0.55 (1.25)

Notes: BSAP = bone specific alkaline phosphatase; P1NP = procollagen type 1 N-propeptide; CTX = carboxylterminal cross-linking. Telopeptide of bone collagen; PTH = parathyroid hormone; Ca = calcium; SHBG = sex hormone binding globulin.

A total of 34 patients had a history of prior fracture, (1 coccyx/sacrum, 16 upper extremity, 16 lower extremity, 4 clavicle/rib and 1 skull). History of maternal hip fracture was reported by 5.8% of patients, with no history for 83.1% of patients and unknown for 11.1%. The total percentage of patients presenting with risk factors for falls at baseline was assessed, 12.9% reported poor vision, 4.9% dizziness, 4.4% difficulty in walking, 1.8% difficulties with body balance, and 1.3% cognitive impairment.

## Discussion

The purpose of this study is to understand underlying mechanisms that could account for increased fracture risk in T2DM patients on RSG. The pattern of these fractures is not typical of fractures seen in a typical cohort postmenopausal women with osteoporosis in whom fractures of the central skeleton (spine and hip) are more common. Loss of bone mass, which has been demonstrated in preclinical and clinical studies occurs, but these studies do not fully explain atypical sites of the fractures. A broad range of factors such as potential decrease in bone quality due to post-translational glycation of bone collagen proteins and clinical conditions such as visual impairment and/or increase in risk of falls, and lifestyle, may act synergistically on diabetic women to increase the propensity for fractures.^17^^–^^19^ In ovariectomized rats, RSG significantly reduced BMD at the whole body, lumbar spine, and proximal tibia when compared to controls, mainly due to trabecular bone loss. A 14 week trial in 50 postmenopausal non-diabetic women showed a significant reduction in total hip BMD and bone formation markers (P1NP and osteocalcin) in RSG versus placebo cohorts.^20^ A redirection of the pathway of stem cells from the osteoblast pathway to adipocyte lineage has been suggested as a potential mechanism by which bone formation could be reduced.^21^ The present study will provide information on the action of RSG and MET on multiple indices of bone mass, skeletal micro- and macro-anatomy, estimated strength, and serum markers of bone remodeling and metabolism. The design in a population of postmenopausal women with T2DM was controlled, randomized, and double-blind study. A 24-week open-label follow-up period, in which all participants discontinued their double-blind study medication and were treated with open-label MET, will allow the assessment of whether any effect of RSG on bone density and structure and serum bone markers resolved after discontinuation.

The study focused upon the femoral neck, a site of substantial cortical bone since the fractures that are being described in these patients treated with RSG were at sites comprised predominantly of cortical bone (upper humerus, hand and foot). To obtain a more complete picture of how TZDs’ effect skeletal microstructure, imaging techniques such as QCT and hrMRI were employed. This study had several limitations. There are not well accepted techniques to determine bone mass and structure at the distal limbs, sites where fractures were reported in clinical studies with RSG. Femoral neck BMD was chosen as the primary endpoint as changes can be compared with reference databases and clinical implications can be derived from changes over time. The novel image techniques were exploratory and there are not validated reference ranges for its values. The study was neither designed nor powered to demonstrate any differences between RSG versus MET in any of the parameters tested. The study population was limited to postmenopausal women, as they were at highest risk of bone loss and fracture and the results may be limited to that age and gender. Also, the 6 month extension where all subjects are placed on open-label MET may be too short to determine the reversibility of any effects noted with RSG. By its nature and design, this was a hypothesis-generating study.

Strengths of the study included randomized double-blind design, multiple simultaneous assessments of state-of-the-art imaging technology and laboratory parameters of bone metabolism, remodeling and hormonal function. The population selected was one that is at the highest risk for fracture, and was therefore clinically relevant. The baseline findings were typical of postmenopausal women with T2DM, making the findings to be obtained applicable to this population.

## Conclusion

This is the first prospective double-blind, randomized, active-controlled clinical trial to simultaneously evaluate the effect of a RSG on bone mass, micro- and macro-structure, and laboratory parameters reflecting bone remodeling mechanisms in a population of postmenopausal women with T2DM. The characterization of the population at baseline is concordant with women at risk for bone loss and subsequent fracture. Understanding potential effects of RSG on the indices to be measured will increase our ability to develop bone protective strategies in patients with T2DM requiring antidiabetic medications.

**Funding**

This clinical study (protocol number AVD111179) is funded by GlaxoSmithKline (GSK).
